# Supplementary material for: A structural model of flagellar filament switching across multiple bacterial species
Source: Nat Commun. 2017 Oct 16;8:960. doi: 10.1038/s41467-017-01075-5 (PMC5643327; doi:10.1038/s41467-017-01075-5)
Supplement: Supplementary file 1 — Supplementary Information [file 41467_2017_1075_MOESM1_ESM.pdf]

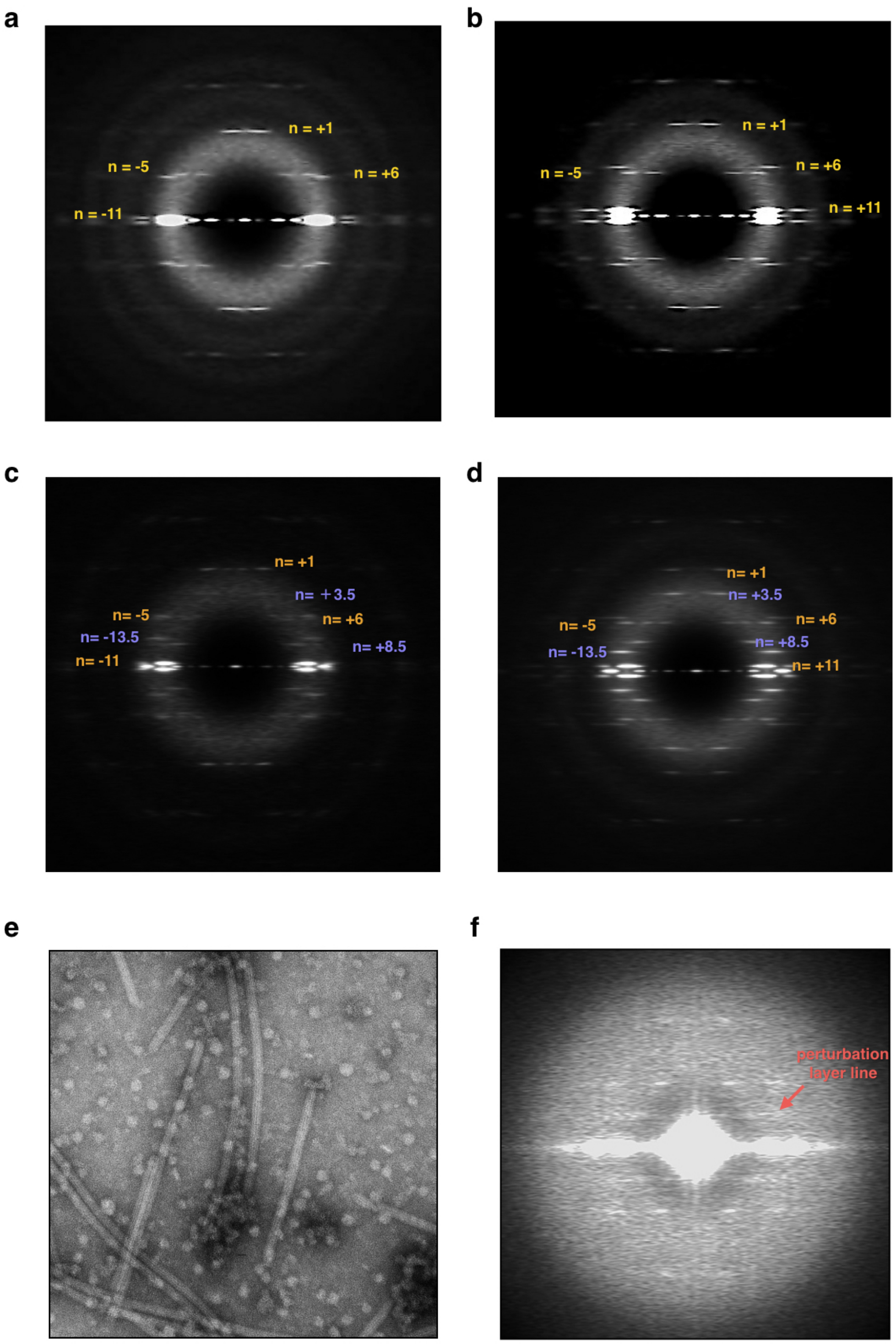

Supplementary Figure 1. Power spectra of flagellar filaments. The power spectra of raw image stack of L- (a) and R-type (b) straight filaments of *B. subtilis*. The power spectra of raw image stack of L- (c) and R-type (d) straight filaments of *P. aeruginosa*. (e) An image of negatively stained wild-type flagellar filaments of *P. aeruginosa*, and the corresponding averaged power spectrum from these filaments showing that the non-helical perturbation observed in mutants also exists in the wild-type.

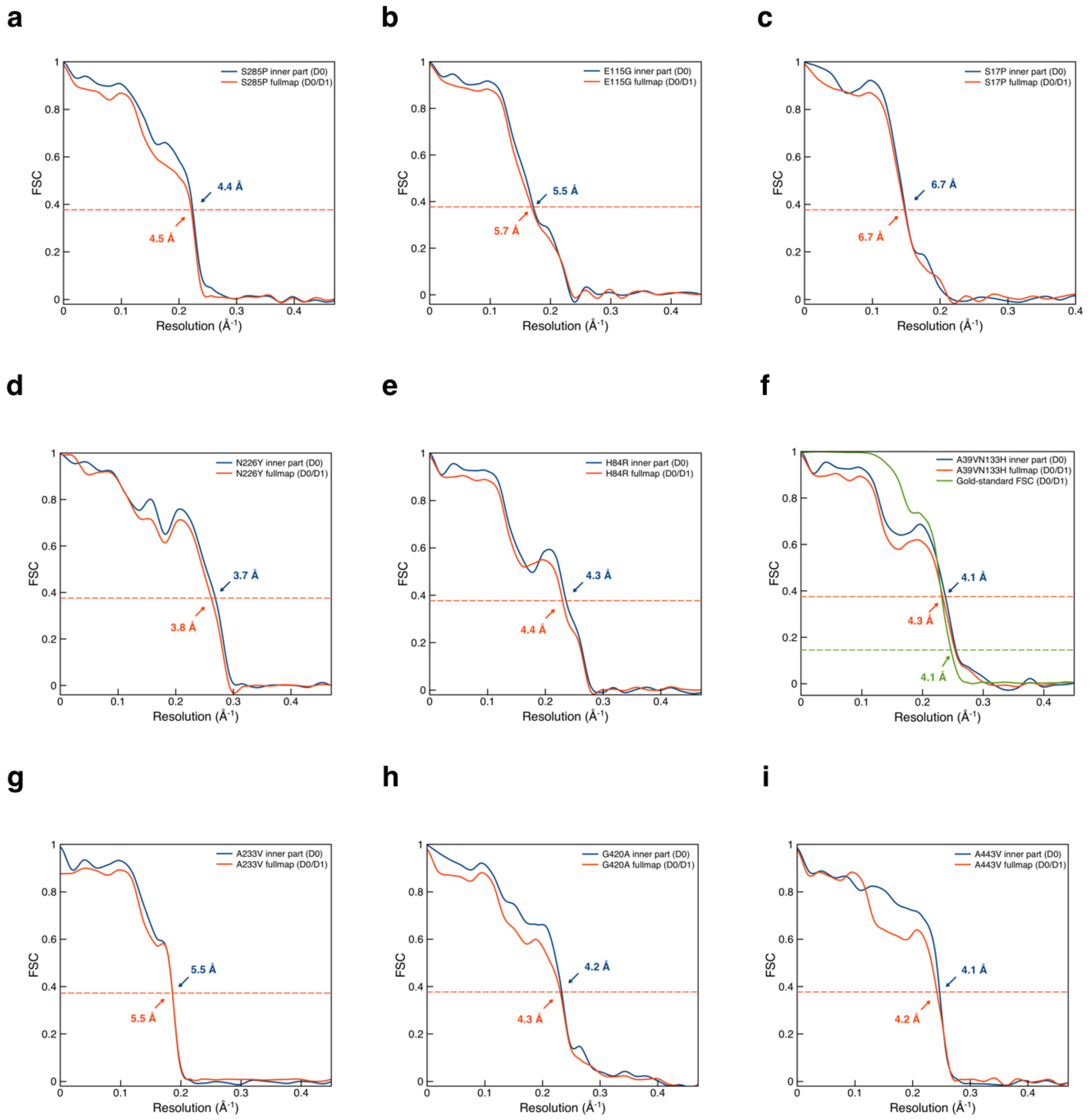

Supplementary Figure 2. Resolution of the flagellar filament structures of *B. subtilis* (a-g) *and P. aeruginosa* (h, i), derived from Fourier Shell Correlation (FSC) calculation. The red and blue lines are FSC of the full map and the D0 core, respectively, calculated between the refined atomic model and the map. The resolution in these plots is estimated using FSC=0.38, which is sqrt(0.143). A "gold standard" FSC between two half maps was also calculated for the A39VN133H dataset (f, green line) yielding a resolution of 4.1 Å at FSC=0.143. This suggests that the model:map FSC curves provide a similar estimate of resolution as the more conventional map:map FSC, and if anything are more conservative.

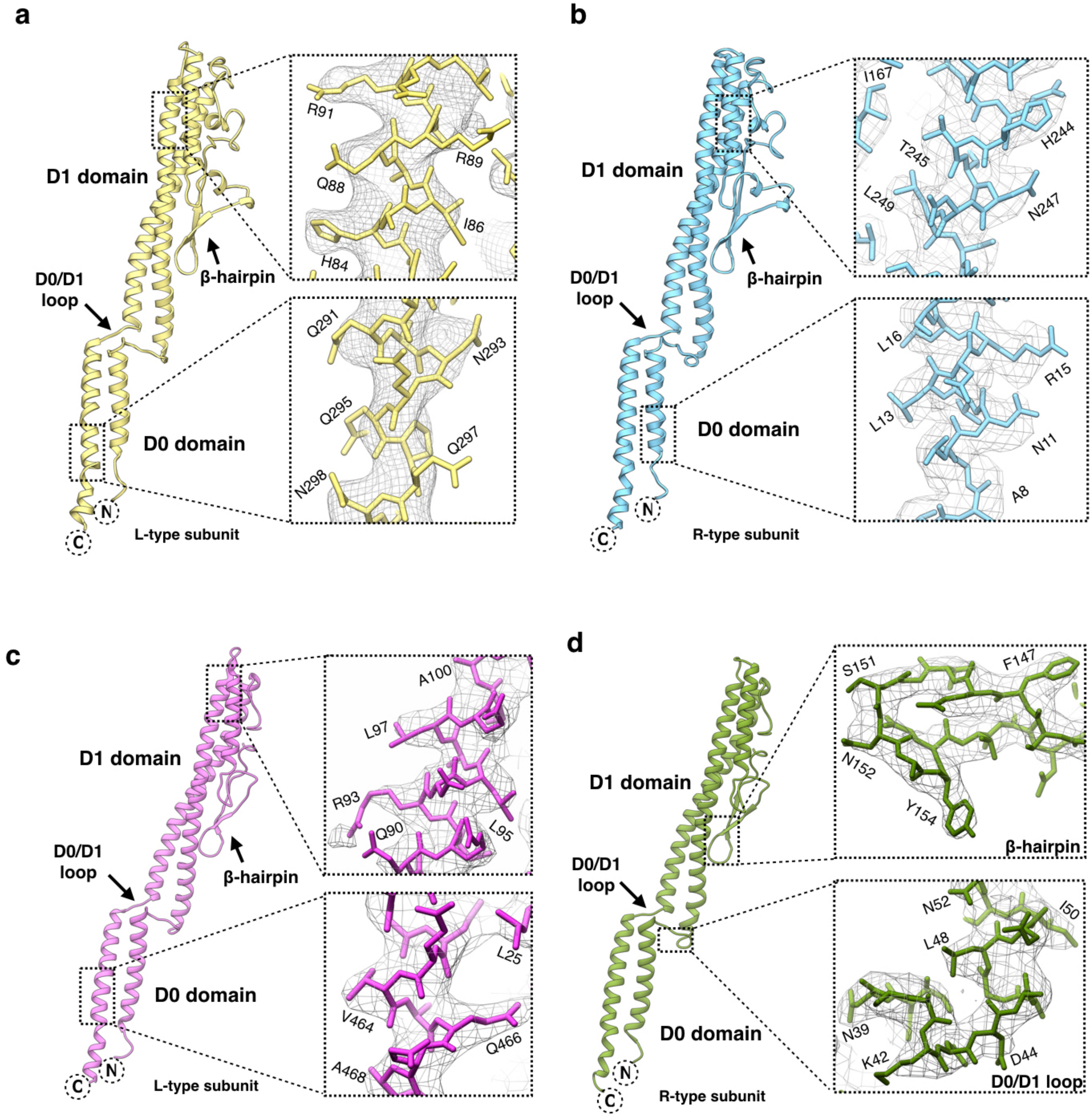

Supplementary Figure 3 The structure of a single flagellin subunit and its corresponding map density in *B. subtilis* and *P. aeruginosa*. **(a)** L-type subunit (S285P) of *B. subtilis* **(b)** R-type subunit (N226Y) of *B. subtilis* **(c)** L-type subunit (G420A) of *P. aeruginosa* **(d)** R-type subunit (A443V) of *P. aeruginosa*

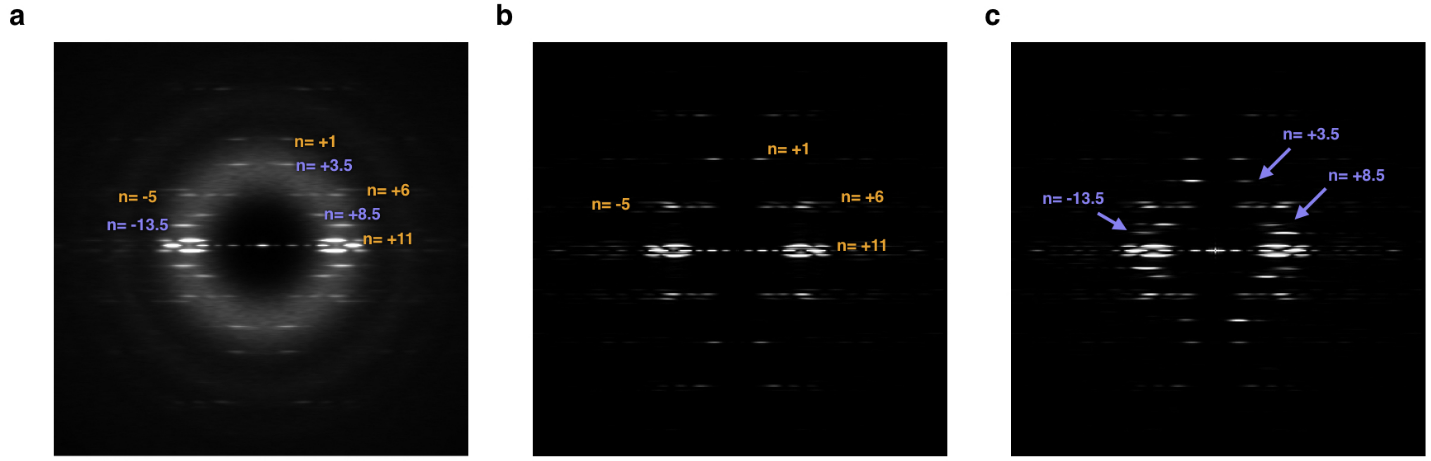

Supplementary Figure 4 power spectra of *P. aeruginosa* filaments. **(a)** The power spectrum of raw filament images of *P. aeruginosa.* **(b)** The power spectrum of the reconstructed volume projection, considering one subunit as the asymmetrical unit (D0 and D1 domains) **(c)** The power spectrum of the reconstructed volume projection, considering 22 subunits as the asymmetrical unit (D2 and D3 domains)

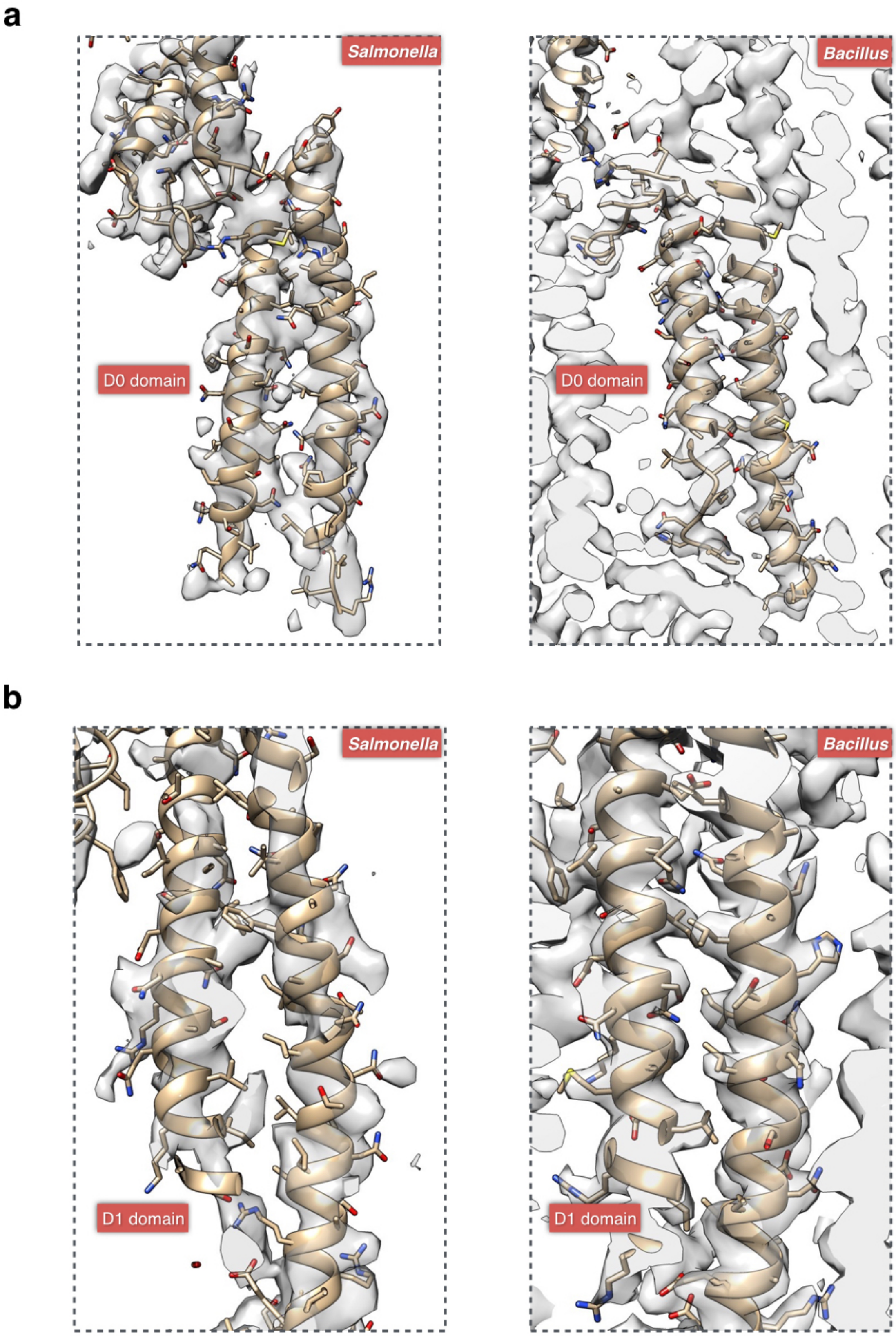

Supplementary Figure 5. The density maps comparison between *Salmonella* (EMD-1641) and *Bacillus* (N226Y). Corresponding cryo-EM map of D0 and D1 domains with similar threshold are shown in (a) and (b). The atomic models were aligned to the maps.

```
Bacillus_subtilis             1 MR--INHNIAALNTLNRLSSNNSASQKNMEKLSSGLRINRAGDDAAGLAISEKMRGQIRGLEMASKNSQDGISLIQT 75
Clostridium_tetani            1 MI--INHNLNAMNAHRQMGINIGNAGKATEKLSSGLRINRAGDDAAGLAISEKMRGQIRGLNQASRNAQDGISLIQT 75
Listeria_monocytogenes        1 MK--VNTNIISLKTQEYLRKNNEGMTQAQERLASGKRINSSLDDAAGLAVVTRMNVKSTGLDAASKNSSMGIDLLQT 75
Streptococcus_pneumoniae      1 MR--IGTNVLSMNARQSLYENEKRMNVAMEHLATGKKLNHASDNPANVAIVTRMYARASGMRVAIRNNEDAISMLRT 75
E_coli                        1 MAQVINTNSLSLITQNNINKNQSALSSSIERLSSGLRINSAKDDAAGQAIANRFTSNIKGLTQAARNANDGISVAQT 77
Helicobacter_pylori           1 MSFRINTNIAALTSHAVGVQNNRDLSSSLEKLSSGLRINKAADDSSGMAIADSLRSQSANLGQAIRNANDAIGMVQT 77
Salmonella_enterica           1 MAQVINTNSLSLLTQNNLNKSQSALGTAIERLSSGLRINSAKDDAAGQAIANRFTANIKGLTQASRNANDGISIAQT 77
Pseudomonas_aeruginosa        1 MALTVNTNIASLNTQRNLNASSNDLNTSLQRLTTGYRINSAKDDAAGLQISNRLSNQISGLNVATRNANDGISLAQT 77

Bacillus_subtilis            76 AEGALTETHAILQRVRELVVQAGNTGTQDKATDLQSIQDEISALTDEIDGISNRTEFNGKKLLDGTYKVDTATPANQ 152
Clostridium_tetani           76 AEGALNETHAILHRMKELTVQAANDTNVT--VDKDNLQLEIKELQSEINRIASQTQFNTKTLLNGSLSK-------- 142
Listeria_monocytogenes       76 ADSALSSMSSILQRMRQLAVQSSNGSFSD--EDRKQYTAEFGSLIKELDHVADTTNYNNIKLLDQTATGA------A 144
Streptococcus_pneumoniae     76 AEAALQTVTNILQRMFDLAVQSANGTNSN--KNRDLLSKEFQSLIEQSGYIGEMTEFNDLSVFDGQNRP-------- 142
E_coli                       78 TEGALSEINNNLQRVRELTVQATTGTNSE--SDLSSIQDEIKSRLDEIDRVSGQTQFNGVNVLAKNGS--------- 143
Helicobacter_pylori          78 ADKAMDEQIKILDTIKTKAVQAAQDGQTL--ESRRALQSDIQRLLEELDNIANTTSFNGQQMLSGSFS--------- 143
Salmonella_enterica          78 TEGALNEINNNLQRVRELAVQSANSTNSQ--SDLDSIQAEITQRLNEIDRVSGQTQFNGVKVLAQDNT--------- 143
Pseudomonas_aeruginosa       78 AEGALQQSTNILQRIRDLALQSANGSNSD--ADRAALQKEVAAQQAELTRISDTTTFGGRKLLDGSFGT-------- 144

Bacillus_subtilis           153 KNLVFQIGANATQQISVNIEDMGADALGIKEA---DG---------------------------------------- 186
Clostridium_tetani          143 SALTFQIGANAGQTITLTIGDMTTSGLKLASI---NI---------------------------------------- 176
Listeria_monocytogenes      145 TQVSIQASDKANDLINIDLFNAKGLSAGT--ITL---GSGS-------T-VA--GY--------------------- 185
Streptococcus_pneumoniae    143 --ITLD---DIGHTINMTKHIPPS----------------------------------------------------- 161
E_coli                      144 --MKIQVGANDNQTITIDLKQIDAKTLGLDGFSV---KNNDTVTTSA-P-VT--AFGATTTNNIKLT------GITL 205
Helicobacter_pylori         144 -NKEFQIGAYSNTTVKASIGSTSSDKIGHVRMETSSFSGEGMLASAAAQNLTEVGLNFKQ---VNGVNDYKIETVRI 216
Salmonella_enterica         144 --LTIQVGANDGETIDIDLKQINSQTLGLDTLNV---QQKYKVSDTAAT-VT--GYADTT---IALD------NSTF 203
Pseudomonas_aeruginosa      145 --TSFQVGSNAYETIDISLQNASASAIGSYQVGS---NGAGTVASVAGT-ATASGIASGT---VNLV------GGGQ 206

Bacillus_subtilis           187 ----------------------SI---------------------------------AALHSVN------------- 195
Clostridium_tetani          177 ---------------------------------------------------------GG------------------ 178
Listeria_monocytogenes          -----------------------------------------------------------------------------
Streptococcus_pneumoniae        -----------------------------------------------------------------------------
E_coli                      206 ST----EAATDTGGTNPASIEGV--YTDNGNDYYAK--ITGGDNDG-KY-YA-------------VTVAN-DGTVTM 258
Helicobacter_pylori         217 ST----SAGTGIGALSE-IINRFSNTLGVRASY--NVMATGGTPVQSGTVRELTINGVEIGTVNDVHKND--A---- 280
Salmonella_enterica         204 -K----ASATGLGGTDQ-KIDGDLKFDDTTGKYYAKVTVTGGTGKD-GY-YE-------------VSVDKTNGEVTL 259
Pseudomonas_aeruginosa      207 VKNIAIAAGDSAKAIAE-KMDGAIPNLSARARTVFTADVSGVTGGS-LN-FD-------------VTVGS--NTVSL 265

Bacillus_subtilis           196 ------------------DLDV-------------------------------------------TKFADNA----- 206
Clostridium_tetani              -----------------------------------------------------------------------------
Listeria_monocytogenes          -----------------------------------------------------------------------------
Streptococcus_pneumoniae        -----------------------------------------------------------------------------
E_coli                      259 ATGATANAT----------VTD-----ANTTKATTITSGGTPVQIDNTAGSATANLGAVS--LVKL--QDSKGNDTD 316
Helicobacter_pylori         281 ----------------DGRLTNAINSVKDRT-------------GVEASLDIQGRINLHSIDGRAISVHAASA---S 325
Salmonella_enterica         260 AGGATSPLTGGLPATATEDVKNVQVANADLT-------------EAKAALTAAGVTGTAS--VVKMSYTDNNGKTID 321
Pseudomonas_aeruginosa      266 AGV-----------TSTQDLADQLNSNSSKL-------------GITASINDKGVLTITSATGENVKFGAQTGTATA 318

Bacillus_subtilis           207 ------------------------------------------------A---------------------------- 207
Clostridium_tetani          179 ------------------------------------------------A---------------------------- 179
Listeria_monocytogenes          -----------------------------------------------------------------------------
Streptococcus_pneumoniae    162 -------------------------------------------------------------PTQ------------- 164
E_coli                      317 TYAL-----------------KDTNGNL-----------------YAADVNETTGAVSVKTITYTDSSGAASSPTAV 359
Helicobacter_pylori         326 G-QVFGGGNFAGISGTQHAVIGRLTLTRTDARDIIVSGVNFSHVGFHSAQGVAEYTVNLRAVRGIFDANVA------ 395
Salmonella_enterica         322 G-GL-----------------AVKVGDD-----------------YYSATQNKDGSISINTTKYTADDGTSKTAL-N 362
Pseudomonas_aeruginosa      319 G-QVA--VKVQGSDGKFEAAAKNVVAAG-----------------TAATTTIVTGYVQLNSPTAYSVSGTGTQ---- 371

Bacillus_subtilis           208 ------------------------------------------DTADIGFDAQLKVVDEAINQVSSQRAKLGAVQNRL 242
Clostridium_tetani          180 ------------------------------------------KGA-ASISSQLKTIDTALNEVSKERAKLGANQNRL 213
Listeria_monocytogenes      186 -------------------------------------SALSVADA-DSSQQATEAIDELINNISNGRALLGAGMSRL 224
Streptococcus_pneumoniae    165 -------------------------------------HDIKISTE-QEARTAIRKIEEALQNVSLHRADLGAMINRL 203
E_coli                      360 KLGGDDGKTEVVDIDGKTYDSADLNGGNLQTGLTAGGEALTAVAN-GKTTDPLKALDDAIASVDKFRSSLGAVQNRL 435
Helicobacter_pylori         396 -----------------SAAGANANGAQAETNSQG--IGAGVTSL-KGAMIVMDMADSARTQLDKIRSDMGSVQMEL 452
Salmonella_enterica         363 KLGGADGKTEVVSIGGKTYAASKAEGHNFKAQ-----PDLAEAAA-TTTENPLQKIDAALAQVDTLRSDLGAVQNRF 433
Pseudomonas_aeruginosa      372 --------------------ASQVFGNASAAQKSS-VASVDISTA-DGAQNAIAVVDNALAAIDAQRADLGAVQNRF 426

Bacillus_subtilis           243 EHTINNLSASGENLTAAESRIRDVDMAKEMSEFTKNNILSQASQAMLAQANQQPQNVLQLL-R               304
Clostridium_tetani          214 EHTIANVNNASENLQAAESRVRDVDMAKEMMNFSKNNILQQAAQAMLAQANQAPQGILQLL-R               275
Listeria_monocytogenes      225 SYNVSNVNNQSIATKASASSIEDADMAAEMSEMTKYKILTQTSISMLSQANQTPQMLTQLINS               287
Streptococcus_pneumoniae    204 QFNIENLNNQSMALTDAASLIEDADMAQEMSDFLKFKLLTEVALSMVSQANQIPQMVSKLLQS               266
E_coli                      436 DSAVTNLNNTTTNLSEAQSRIQDADYATEVSNMSKAQIIQQAGNSVLAKANQVPQQVLSLLQG               498
Helicobacter_pylori         453 VTTINNISVTQVNVKAAESQIRDVDFAEESANFSKYNILAQSGSFAMAQANAVQQNVLRLL-Q               514
Salmonella_enterica         434 NSAITNLGNTVNNLTSARSRIEDSDYATEVSNMSRAQILQQAGTSVLAQANQVPQNVLSLL-R               495
Pseudomonas_aeruginosa      427 KNTIDNLTNISENATNARSRIKDTDFAAETAALSKNQVLQQAGTAILAQANQLPQAVLSLL-R               488
```

Supplementary Figure 6. Alignments of the flagellin amino acid sequence from four Gram-positive bacteria and four Gram-negative bacteria. This alignment contains full sequences with D2 and D3 domains. Dark blue indicates higher sequence identities among species and light blue indicates intermediate sequence identities among species.

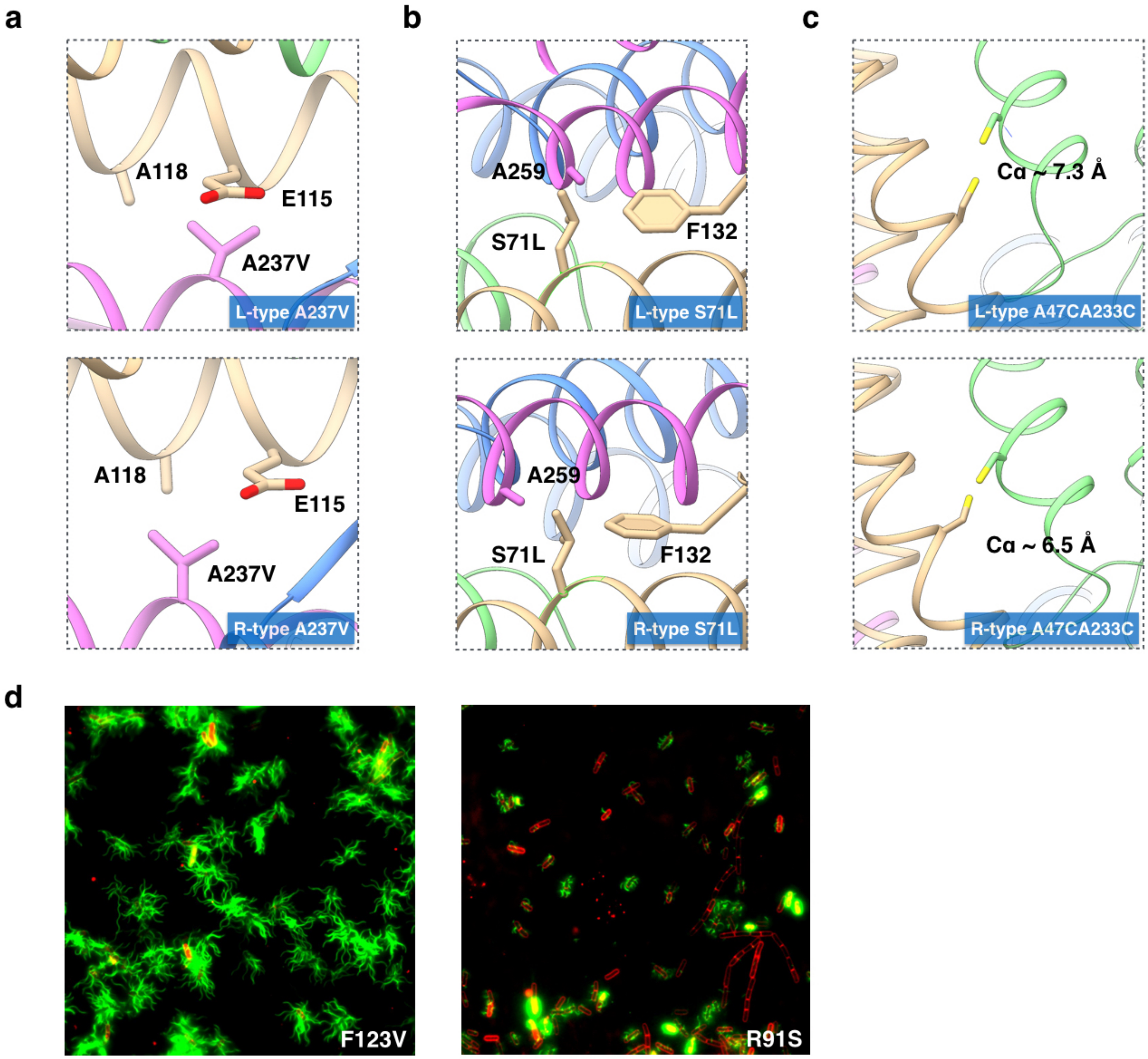

Supplementary Figure 7. Predicted mutation sites in *B. subtilis*. (a-c) Straight filaments S285P and N226Y structures were used as the modeling template, and the resulting interfaces are shown in (a) A237V (b) S71L and (c) A47CA233C. (d) Fluorescence images of three mutant filaments: F123V and R91S

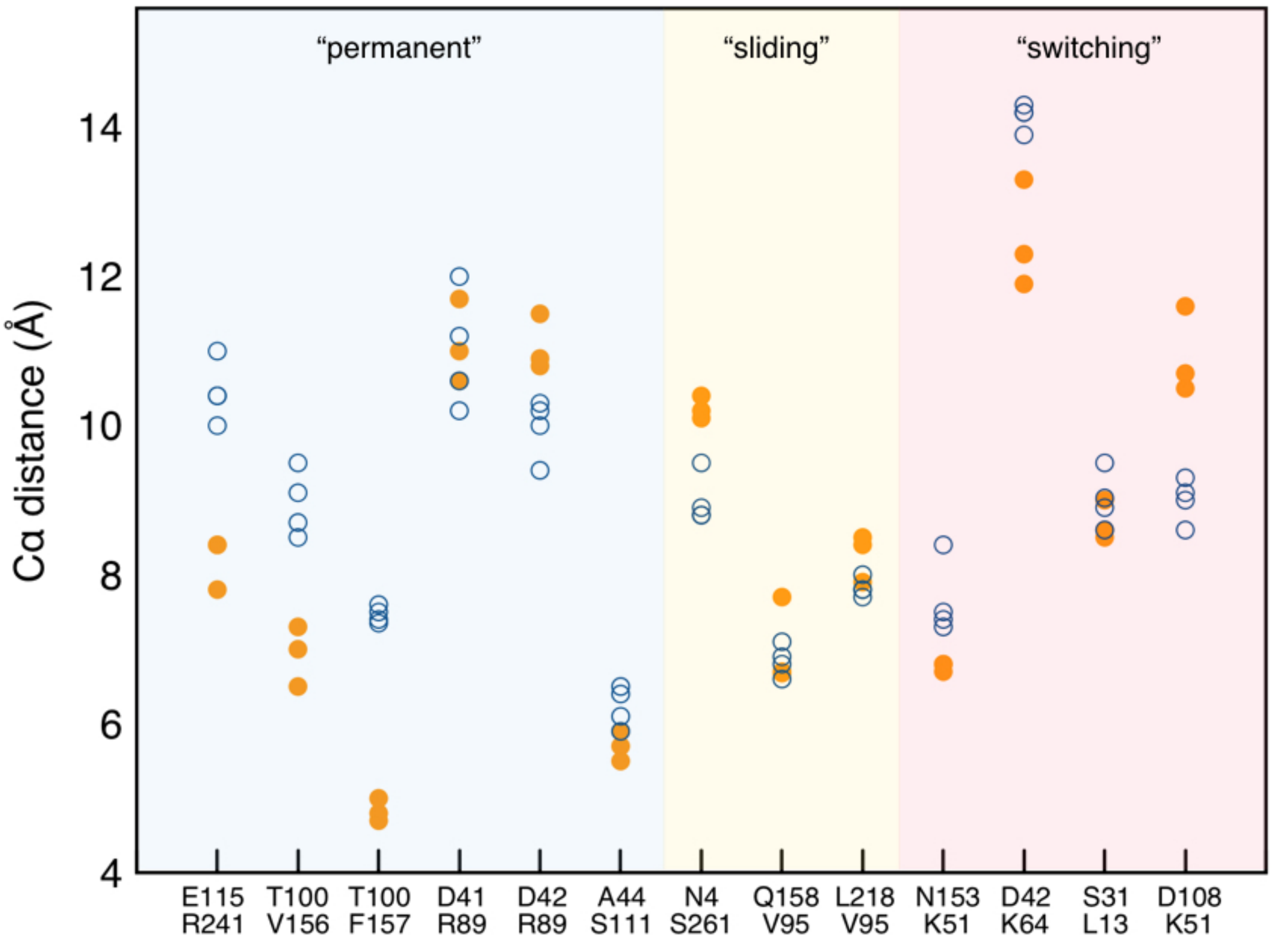

Supplementary Figure 8. Mapping three types of interactions in *Salmonella* onto seven *Bacillus* mutants. Three types of interactions were described in *Salmonella*, called “permanent”, “sliding” and “switching” interactions (Kitao et al., 2006), and many *Salmonella* residues are involved in those interactions. The corresponding residues in *Bacillus* can be identified and mapped based on the sequence alignment. Here we are showing the Cα distances of the corresponding *Bacillus* residues that belong to those three types of interactions. The orange solid circles are the three L-type straight mutants (E115G, S17P and S285P), and the blue empty circles are the four R-type straight mutants (H84R, N226Y, A233V, and A39VN133H).

Supplementary Table 1: Subunit-subunit interfacial area along different helices

Mutation site(s)	5-start (Å^2)	11-start (Å^2)	6-start (Å^2)	16-start (Å^2)
B. subtilis				
S285P	1850	1954	552	238
E115G	1823	1810	485	237
S17P	1893	1833	574	265
N226Y	1819	1985	683	278
A39VN133H	1673	1874	596	275
H84R	1671	1924	516	330
A233V	1779	1867	571	271
P. aeruginosa				
G420A	1800	1630	495	211
A443V	1758	1922	542	286

Supplementary Table 2 Strains and mutations

Strain	Genotype	
3610	Wild type	
DS1677	*Δhag*	(Kearns and Losick, 2005)
DS1919	*Δhag amyE::*P_{hag}-*hag*^{T209C} *spec*	(Blair *et al.*, 2008)
DK29	$P_{fla/che}$Ω*Physpank* fla/che operon *kan*	(Guttenplan *et al.*, 2013)
DK620	[PY79] *Δhag sfp*^{0} *swrA*^{fs}	
DK1343	*Δhag amyE*::P_{hag}-*hag*^{S17PT209C} *spec*	
DK1362	*Δhag* amy*E*::P_{hag}-*hag*^{H84RT209C} *spec*	
DK2750	*Δhag amyE*::P_{hag}-*hag*^{T209CN226Y} *spec*	
DK2751	*Δhag amyE*::P_{hag}-*hag*^{A39VN133HT209C} *spec*	
DK2790	*comI*^{Q12L} *Δhag*	
DK2956	*Δhag amyE*::P_{hag}-*hag*^{S285PT209C} *spec*	
DK2977	*Δhag amyE*::P_{hag}-*hag*^{E115GT209C} *spec*	
DK3400	*Δhag amyE*::P_{hag}-*hag*^{A233VT209C} *spec*	
DK3965	*Δhag* amy*E*::P_{hag}-*hag*^{H84RT209C} *spec* $P_{fla/che}$Ω*Physpank fla/che operon kan*	
DK4152	*Δhag amyE*::P_{hag}-*hag*^{S17PT209C} *spec* $P_{fla/che}$Ω*Physpank fla/che operon kan*	
DK4153	*Δhag amyE*::P_{hag}-*hag*^{E115GT209C} *spec* $P_{fla/che}$ΩP*hyspank fla/che operon kan*	
DK4229	*Δhag amyE*::P_{hag}-*hag*^{T209CN226Y} *spec* $P_{fla/che}$ΩP*hyspank fla/che operon kan*	
DK4230	*Δhag amyE*::P_{hag}-*hag*^{A39VN133HT209C} *spec* $P_{fla/che}$ΩP*hyspank fla/che operon kan*	
DK4324	*Δhag amyE*::P_{hag}-*hag*^{A233VT209C} *spec* $P_{fla/che}$ΩP*hyspank fla/che operon kan*	
DK4399	*Δhag amyE*::P_{hag}-*hag*^{S285PT209C} *spec* $P_{fla/che}$ΩP*hyspank fla/che operon kan*	
DK4859	*comI*^{Q12L} *Δhag aprE*::P_{hag}-*hag*^{S31PT209C} *kan*	
DK4860	*comI*^{Q12L} *Δhag aprE*::P_{hag}-*hag*^{S71LT209C} *kan*	
DK4864	*comI*^{Q12L} *Δhag aprE*::P_{hag}-*hag*^{T209CA237V} *kan*	
DK4879	*comI*^{Q12L} *Δhag aprE*::P_{hag}-*hag*^{T209CF123V} *kan*	
DK4884	*comI*^{Q12L} *Δhag aprE*::P_{hag}-*hag*^{R91ST209C} *kan*	
DK4894	*comI*^{Q12L} *Δhag aprE*::P_{hag}-*hag*^{A47CT209CA233C} *kan*	
PY79	*sfp*^{0} *swrA*^{fs}	

Supplementary Table 3 List of used primers

Primers	Genotype
953	GGAGTGTCAAGAATGTTTGCAAAAC
1008	TGCAGCCGCTGAAGAATATGGCA
1009	TGCCGTATTGATAGAGAXAGAGGA
1115	AGGAGGAATTCTTTAGAATCGCACCTTAGCTGGTG
1116	CTCCTCTCGAGGCCAGAACCAGCAGCGGAGCCAGCGGAACCTGATCCCGAAGCGACTG CTTCG
1117	AGGAGGAATTCCCTTACCTGATTGATATGCTGTAATG
1118	CTCCTCTCGAGGCCAGAACCAGCAGCGGAGCCAGCGGAACCATCCTTTTCTTTAAGCTG ACGCTCC
3251	CTCACTAAAGGGAACAAAAGCTGG
3459	GCTAATACGACTCACTATAGGGTAATGATGTAGCCGGGAGGA
4439	CGTTTAGAAAGCACTGCAACA
4440	CGCCATCGTAAAGATTAACGT
4893	TACCACGATGGCTGCCACTA
4894	GTACTTGCCACCACATCATAA
4895	TGTTGCAGTGCTTTCTAAACGGCGTTATCCAGCGATGTGAT
4897	ACGTTAATCTTTACGATGGCGACGACTCACTATAGGGCGAATTG
4932	CTTTTGTTCCCTTTAGTGAGCTGGCAACGCCAAGGTCTTTTTTAAAA
5518	GAAACTTCCTTCAGGTCTTCGCATCAAC
5519	GAAGGAAGTTTCTCCATGTTCTTTTG
5520	GAATCCTTCTTATCCAAACAGCTGAG
5521	GATAAGAAGGATTCCGTCTTGAGAG
5522	GTTAGTGAGCTAGTTGTTCAAGCTGG
5523	CTCACTAACACGTTGAAGGATCGCATG
5524	GAAGTCAATGGTAAGAAATTGCTCG
5525	CATTGACTTCTGTACGATTTGAATAC
5526	GGTGTGGTACAAAATCGTCTAGAGCAC
5527	GTACCACACCAAGCTTAGCACGTTGAG
5528	CTTTGCATCTCTGAAAAAATGAGAGG
5529	GATGCAAAGACCTGCTGCGTCATCTC
5530	CGTTGCAAGCTTGGTGCGGTACAAAATC
5531	GCTTGCAACGTTGAGAAGAAACTTGG

Reference

Kitao, A., Yonekura, K., Maki-Yonekura, S., Samatey, F.A., Imada, K., Namba, K., and Go, N. (2006). Switch interactions control energy frustration and multiple flagellar filament structures. Proc Natl Acad Sci U S A *103*, 4894-4899.
